# Association of Age and Structural Brain Changes With Functional Connectivity and Executive Function in a Middle-Aged to Older Population-Based Cohort

**DOI:** 10.3389/fnagi.2022.782738

**Published:** 2022-02-25

**Authors:** Maximilian Schulz, Carola Mayer, Eckhard Schlemm, Benedikt M. Frey, Caroline Malherbe, Marvin Petersen, Jürgen Gallinat, Simone Kühn, Jens Fiehler, Uta Hanning, Raphael Twerenbold, Christian Gerloff, Bastian Cheng, Götz Thomalla

**Affiliations:** ^1^Department of Neurology, University Medical Center Hamburg-Eppendorf, Hamburg, Germany; ^2^Institute of Computational Neuroscience, University Medical Center Hamburg-Eppendorf, Hamburg, Germany; ^3^Department of Psychiatry and Psychotherapy, University Medical Center Hamburg-Eppendorf, Hamburg, Germany; ^4^Department of Neuroradiological Diagnostics and Intervention, University Medical Center Hamburg-Eppendorf, Hamburg, Germany; ^5^Department of Epidemiology, University Medical Center Hamburg-Eppendorf, Hamburg, Germany; ^6^University Center of Cardiovascular Science, Department of Cardiology, University Heart and Vascular Center Hamburg, University Medical Center Hamburg-Eppendorf, Hamburg, Germany

**Keywords:** age, resting state functional MR imaging, functional connectivity, cortical atrophy, peak width of skeletonized mean diffusivity

## Abstract

Aging is accompanied by structural brain changes that are thought to underlie cognitive decline and dementia. Yet little is known regarding the association between increasing age, structural brain damage, and alterations of functional brain connectivity. The aim of this study was to evaluate whether cortical thickness and white matter damage as markers of age-related structural brain changes are associated with alterations in functional connectivity in non-demented healthy middle-aged to older adults. Therefore, we reconstructed functional connectomes from resting-state functional magnetic resonance imaging (MRI) (rsfMRI) data of 976 subjects from the Hamburg City Health Study, a prospective population-based study including participants aged 45–74 years from the metropolitan region Hamburg, Germany. We performed multiple linear regressions to examine the association of age, cortical thickness, and white matter damage quantified by the peak width of skeletonized mean diffusivity (PSMD) from diffusion tensor imaging on whole-brain network connectivity and four predefined resting state networks (default mode, dorsal, salience, and control network). In a second step, we extracted subnetworks with age-related decreased functional connectivity from these networks and conducted a mediation analysis to test whether the effect of age on these networks is mediated by decreased cortical thickness or PSMD. We observed an independent association of higher age with decreased functional connectivity, while there was no significant association of functional connectivity with cortical thickness or PSMD. Mediation analysis identified cortical thickness as a partial mediator between age and default subnetwork connectivity and functional connectivity within the default subnetwork as a partial mediator between age and executive cognitive function. These results indicate that, on a global scale, functional connectivity is not determined by structural damage in healthy middle-aged to older adults. There is a weak association of higher age with decreased functional connectivity which, for specific subnetworks, appears to be mediated by cortical thickness.

## Introduction

Higher age is accompanied by structural brain alterations, which are thought to give rise to functional abnormalities and decline in cognitive function, even in individuals free of neurodegenerative disease. Pathophysiological processes suspected to cause age-related neurodegeneration are heterogeneous and not well understood, ranging from proteinopathies (Noor et al., 2021) to vascular pathology (Brown and Thore, 2011). Modern brain magnetic resonance imaging (MRI) studies provide an opportunity to probe *in vivo* for changes in structural and functional imaging biomarkers during the aging process. Indeed, researchers observed many neuroimaging measures of brain structure and function that indicate deterioration during adult aging (Hedden and Gabrieli, 2004; Walhovd et al., 2011; Grady, 2012; Chen et al., 2016) with cortical thickness, white matter hyperintensity (WMH) load, and functional connectivity (FC) being the most prominent parameters.

White matter damage visible by hyperintensities (WMH) on T2-weighted MR images represents microvascular ischemic and demyelinating alterations (Chui, 2006; Launer et al., 2006). In the elderly population, white matter damage is considered a risk factor for cognitive impairment (Geppert et al., 2007), impairment in gait (Starr et al., 2003), depression (Dalby et al., 2012), and an increased risk for stroke and dementia (Debette and Markus, 2010). Beyond age, primary contributors to the development of microvascular damage include arteriolosclerosis as well as cardiovascular risk factors such as arterial hypertension, diabetes, and smoking (Stephan et al., 2009; Power et al., 2015; Frey et al., 2019).

Whole-brain decline of cortical thickness with increasing age has been consistently observed in previous research (Fjell and Walhovd, 2010; Hogstrom et al., 2013) and several studies have observed a relationship between cortical volume, cognition, and age (Head et al., 2008; Kirchhoff et al., 2014). Research about the inherent mechanisms describes an increase in glia and small-body neurons and decrease in large cell-body neurons populations throughout aging, contributing to a near constant cell density with diminishing brain volume (Terry et al., 1987; Martínez-Pinilla et al., 2016). Cortical thickness was also found to be associated with the extent of WMH in whole-brain- and region-specific analyses (Du et al., 2005; Seo et al., 2012; Tuladhar et al., 2015), which might be explained by secondary cortical degeneration through damaged white matter tracts (Alosco et al., 2013; Thong et al., 2013; Ter Telgte et al., 2018).

The effect of age on functional brain connectivity is more complex and studies reported heterogeneous effects captured by variety of different measuring methods during the last years. These effects report increase and decrease of mean FC across different cortical regions (Onoda et al., 2012; Vidal-Piñeiro et al., 2014; Zonneveld et al., 2019) as well as a progressive decrease in FC throughout the entire cortex (Farras-Permanyer et al., 2019). Recent studies that focused on alterations of intra- and internetwork connectivity separately report that FC between networks maintains stable or even increases with age, while FC within networks tends to decrease (Betzel et al., 2014; Chan et al., 2014). Looking at network characteristics, higher age was found to be associated with lower system segregation and altered network topology, both throughout the brain and in single-resting state networks (Geerligs et al., 2014).

Functional brain connectivity is considered to be determined by the structural connectome. An accumulating number of studies investigating resting-state fMRI connectivity in the presence of severe white matter damage in form of cerebral small vessel disease (CSVD) or stroke suggest that FC is negatively affected by white matter disconnection (Wu et al., 2015; Kim et al., 2016; Liu et al., 2019) and tract-specific reduction in FC in the context of WMH was reported (Langen et al., 2017). In addition, the hypothesis of secondary cortical degeneration through damaged white matter tracts indicates a direct pathophysiological link between WMH and cortical thickness (Alosco et al., 2013; Thong et al., 2013; Ter Telgte et al., 2018) and since dendrites are involved in interneural communication, it is suggested that alterations in functional interareal connectivity follow that phenomenon (Vieira et al., 2020).

Nevertheless, the question of whether there is an effect of age-related structural changes on FC in a healthy population with low-level white matter damage remains unanswered.

To gain a better insight into this relation, we investigated the association of age, white matter damage, and cortical thinning on FC in a healthy middle-aged to older population. Therefore, we first looked at the influence of age, microstructural white matter damage, and cortical thickness on FC in multiple linear regressions (MLRs). Instead of using WMH load as measure of microstructural alterations, we used the peak width of skeletonized mean diffusivity (PSMD) as a novel and fully automated MRI biomarker. Compared to white matter markers such as white hyperintensity volume, the PSMD eliminates contamination from cerebrospinal fluid (CSF) and the histogram-based approach enhances the ability to characterize subtle, diffuse diseases in the brain such as small vessel disease (Low et al., 2020). In addition, the PSMD is also strongly related to global cognition and has been shown to be closely associated with higher WMH lesion load (Wei et al., 2019). Measures of FC included mean connectivity of the entire cortex, additionally separated in inter- vs. intranetwork connectivity as well as mean connectivity of the default, dorsal, salience, and control network. Since we expected to observe subtle effects in a relatively healthy cohort with a narrow range of age, we restricted our analysis to networks that perform associative, integrative tasks and, according to previous literature, exhibit greater changes with age than sensory systems in the brain (Chan et al., 2014). The default mode network is primarily composed of the medial prefrontal cortex, posterior cingulate cortex, precuneus, and the angular gyrus (Sormaz et al., 2018), while the dorsal attention network is primarily composed of the intraparietal sulcus and frontal eye fields (Fox et al., 2006; Farrant and Uddin, 2015). The default mode network shows greater activity in the task-free state and is mainly related to internally directed cognitions (Buckner et al., 2008); the dorsal attention network displays activation during performing tasks and supports external attention. Both the networks are anticorelated with each other in brain activity. The frontoparietal control network composes the dorsolateral prefrontal cortex and posterior parietal cortex and is considered to flexibly support both the default mode network and the dorsal attention network according to task demands, interpreted as a regulating role (Fornito et al., 2012; Elton and Gao, 2014). All the three functional networks are related to executive function (Niendam et al., 2012; Turner and Spreng, 2012; Dey et al., 2016). The major nodes of the salience network are in, particularly, the anterior insula and dorsal anterior cingulate cortex, which have been implicated as well in various features of executive function, including the orienting of attention (Corbetta and Shulman, 2002) and performance monitoring (Dosenbach et al., 2007). In a second step, we addressed the question if age-related network alterations are driven by specific functional areas and identified within default, dorsal, and salience network disconnected subnetworks that exhibited significant age-related decline in FC. With these subnetworks, representing only the portion of the functional network that is affected by the influence of age, we examined possible mediating effects of the PSMD and cortical thickness on the relationship between age and FC. Since among the different cognitive measurements, executive function seems to be the most vulnerable to age-related decline (Wecker et al., 2000; Verhaeghen and Cerella, 2002; Madden et al., 2017) and we investigated that if mean FC itself is a mediator of the relationship between age and executive cognitive function. We hypothesized that with an increasing age, white matter damage and cortical thickness are associated with an alterations in resting-state FC. Further, we predicted that part of the effect of age on FC is mediated by structural parameters Finally, we expected to observe that the effect of age on executive cognitive function is partially mediated by age-affected FC of the resting-state networks (subnetworks), due to their essential role in executive cognitive function, as described above.

## Materials and Methods

### Study Population

For this study, we analyzed baseline data from the first 1,000 participants of the Hamburg City Health Study (HCHS) who were studied by brain MRI. The HCHS is a single center prospective, epidemiologic cohort study with an emphasis on imaging to improve the identification of individuals at risk for major chronic diseases and to improve early diagnosis and survival. A detailed description of the overall study design has been published separately (Jagodzinski et al., 2020). In brief, 45,000 citizens of the city of Hamburg, Germany, between 45 and 74 years were invited to an extensive baseline evaluation. A subgroup with present cardiovascular risk factors was invited to undergo standardized MRI brain imaging. For this analysis, we included the first 1,000 participants from this subgroup. Participants with brain MRI datasets imaging data of insufficient quality for white matter segmentation and construction of blood oxygenation level dependent (BOLD) signal were excluded from further analysis.

Baseline examinations in the HCHS comprise a set of standardized tests of cognitive function. To probe the association of age-related imaging correlates with executive function, we used data from the Trail Making Test part A and B (TMTA and TMTB) as well as the ratio score between both the tests. As an important covariate for cognitive function, we included information on the years of education. For descriptive purposes, we also included information on cardiovascular risk factors and comorbidities. This study was approved by the Local Ethics Committee of the Landesärztekammer Hamburg (State of Hamburg Chamber of Physicians, PV5131) and a written informed consent was obtained from all the participants.

### Magnetic Resonance Imaging Acquisition

Images were acquired using the 3T Siemens Magnetom Skyra MRI Scanner (Siemens, Erlangen, Germany, United Kingdom). For three-dimensional (3D) T1-weighted anatomical images, rapid acquisition gradient-echo sequence [(Magnetization prepared rapid gradient echo-. sequence (MPRAGE)] was used with the following sequence parameters: repetition time (TR) = 2,500 ms, echo time (TE) = 2.12 ms, 256 axial slices, slice thickness (ST) = 0.94 mm, and in-plane resolution (IPR) = 0.83 mm^2^ × 0.83 mm^2^. 3D T2-weighted fluid-attenuated inversion recovery (FLAIR) images were measured with the following sequence parameters: TR = 4,700 ms, TE = 392 ms, 192 axial slices, ST = 0.9 mm, and IPR = 0.75 mm^2^ × 0.75 mm^2^. We used 5.2 min axial multi-echo echo-planar imaging (EPI) resting-state acquisition during which subjects were instructed to focus on a fixation cross. TR = 2,500 ms, TE = 25 ms, flip angle: 90°, Generalized Autocalibrating Partial Parallel Acquisition (GRAPPA): 2, acquisition matrix: 94 × 94, field of view (FOV): 250 mm^2^ × 250 mm^2^, 40 slices of 3 mm thickness, and 10% slice gap with a reconstructed voxel dimension of 2.7 mm^3^ × 2.7 mm^3^ × 3 mm^3^. A total of 125 volumes were acquired. For the single-shell diffusion-weighted imaging (DWI), 75 axial slices were obtained covering the whole brain with gradients (*b* = 1,000 s/mm^2^) applied along 64 non-collinear directions with the following sequence parameters: TR = 8,500 ms, TE = 75 ms, ST = 2 mm, IPR = 2 mm^2^ × 2 mm^2^, and anterior-posterior phase-encoding direction.

### Peak Width of Skeletonized Mean Diffusivity and Cortical Thickness

The PSMD tool provided at http://www.psmd-marker.com was used to calculate the PSMD (Baykara et al., 2016). The process consists of two steps. First, DWI data including MD were skeletonized *via* tract-based spatial statistics (TBSS) procedure (Smith et al., 2006). Second, the PSMD is a diffusions tensor imaging (DTI)-derived measure based on skeletonization and histogram analysis and is calculated as the difference between the 95th and 5th percentile of MD values within the masked MD skeleton (Low et al., 2020). Mean cortical thickness across the entire cortex was measured on T1-weighted imaging data using the standardized FreeSurfer processing pipeline (version 6.0) (Fischl and Dale, 2000).

### Preprocessing

All the data were preprocessed using fMRIPrep^1^, a fully reproducible, preconfigured preprocessing pipeline for functional MRI, which works with several tools such as the Advanced Normalization Tools (ANTs)^2^, and FreeSurfer version 6.0.^3^ The preprocessing of the structural MR images included brain extraction, tissue segmentation, non-linear spatial normalization, surface preprocessing, and refinement of the brain mask. Functional preprocessing steps included BOLD reference image estimation, head-motion correction, susceptibility distortion correction (SDC), transformation of BOLD in native space, EPI to T1W registration, and resampling BOLD runs onto the Montreal National Institute (MNI) standard spaces.

### Denoising

The denoising procedure was done with the XCP Imaging Pipeline (xcpEngine)^4^ using independent component analysis (ICA)-based strategy for automatic removal of motion artifacts (ICA-Aroma) with the global signal regression to reduce the influence of motion on the data. ICA-AROMA uses four characteristics to determine whether each component corresponds to a signal or noise. The first two characteristics are spatial properties of the signal source: (1) the portion of the source that falls within a CSF compartment and (2) the portion of the source that falls along the edge or periphery of the brain. The other characteristics are derived from the time series of the source: (3) its maximum robust correlation with the time series, derived from realignment parameters and (4) its high-frequency spectral content. ICA-AROMA comprises two denoising steps. The first denoising step occurs immediately after classification. All the component time series (signal and noise) are included as predictors in the linear model and the residual BOLD time series is obtained *via* partial regression of only the noise time series. A second confound regression step occurs after temporal filtering (high-pass: 0.01, low-pass: 0.08), wherein mean signals from white matter and CSF and the global signal were regressed from the data (Ciric et al., 2017).

### Network Construction and Measurement of Functional Connectivity

The reconstructed functional whole brain comprised nodes representing brain regions and edges as inter-regional resting-state FC. To define the network nodes, we divided the brain into 200 contiguous and uniform regions of interests (ROIs) based on local global parcelation of the human cerebral cortex by Schaefer et al. (2018). From these 200 ROIs, four resting-state networks were extracted, which contained the default mode network, dorsal attention network, salience network, and cognitive control network. All the regions of the resting-state networks were determined *a priori* by the Schaefer atlas and were, thus, unique in each resting-state network without overlapping between networks. The individual mean time series were then extracted for each ROI. Finally, absolute values of the Pearson’s correlation coefficients were calculated between each pair of ROIs for each subject, which resulted in a square undirected correlation matrix representing the whole cortex and for each resting-state network. To further denoise spurious interregional correlations, the connectivity matrices were thresholded by preserving a proportion of 50% of the strongest weights. All the other weights and all the weights on the main diagonal (self-connections) were set to zero. Whole-brain and network FC was calculated by taking the mean of the edges between all the nodes in the matrix that were not excluded. In addition, as a measurement of control, we constructed connectivity matrices with absolute values as well as matrices with both the negative and positive correlations, which were unthresholded or preserving a portion of 70, 30, and 10% of the strongest weights (see Supplementary Figures 2, 3).

### Trail Making Test

The Trail Making Test part A (TMTA) is a measure of psychomotor speed (Razzak, 2013). In this test, circles are numbered from 1 to 25 and subjects are required to draw a line connecting the circles in numerical sequence as quickly as possible. The Trail Making Test part B (TMTB) is a measure of executive cognitive control (Reitan, 1958). The TMT-B requires the subject to connect 25 encircled numbers and letters in numerical and alphabetical order, alternating between the numbers and letters, which are randomly distributed in space. The subject is also asked to connect the array of circles as quickly as possible without lifting the pencil. During the tests, the examiner corrects each error immediately (Terada et al., 2013). In both the parts of the trail making test, a shorter completion time is considered a gauge of better cognitive performance and is measured in seconds. In order to adjust for the influence of motor speed and visual search on executive function, we analyzed additionally the ratio score between both the tests (B/A).

### Statistical Analysis

All the statistical analyses were conducted using R version 4.0.2. In an initial step, we used univariate analyses to examine the influence of age on all the imaging measurements and the cognitive TMTA, TMTB as well as the TMT ratio score. To assess the relationship between altered FC as the dependent variable and age, cortical thickness, and the PSMD as predictors, we applied several multiple linear regressions, adjusted for sex and years of education. These linear models were calculated for the mean FC of the brain as a whole and separated for inter- vs. intranetwork connectivity as well as for all the four resting-state networks. To correct for multiple testing, the statistical threshold was set at *p* ≤ 0.007 (0.05/7; based on four networks and three global measurements). All the networks that failed to show a correlation with age were excluded from further analysis. Within each resting-state network, we aimed to identify those edges or subnetworks that significantly correlate with an increasing age. Therefore, we used the network-based statistic (NBS) approach (Zalesky et al., 2010), which addresses multiple comparisons in network statistics. In summary, a *t*-test was performed on each pair of ROIs to test the significance of correlation between FC and an increasing age, based on the values stored in the connectivity matrix of each subject. The connections with a test statistic exceeding a threshold of *t* = 3.2 (*p* = 0.0007) were admitted to the set of suprathreshold links for which a path can be found between any two nodes, thereby forming an interconnected network and are referred to as connected components in graph theory. The assumption is that the topological configuration of a putative experimental effect is well represented by a component and is not confined to a single connection or distributed across multiple connections that are isolated from each other. To evaluate the significance for each component, a familywise error (FWE)-corrected *p*-value was then ascribed to each component based on its size using permutation testing. The NBS was then applied to the randomized data and the size of the largest component was recorded. A total of 5,000 permutations were generated to estimate the null distribution of the maximal component size. Subsequently, the corrected *p*-value for an observed component found in the original data was calculated as the proportion of permutations for which the maximal component size was greater than or equal to the observed component, ensuring control of the FWE rate. To better visualize the resting-state networks and their significant disconnected subnetworks correlated with age, we used the BrainNet Viewer (Xia et al., 2013). Finally, mediation analyses using the Lavaan package (Rosseel, 2012) were conducted to further interrogate first possible mediation effects of the PSMD and cortical thickness between age and FC in the studied subnetworks and second a possible mediation effect of FC between age and executive cognitive function. Therefore, we used non-parametric bootstrapping with 10,000 iterations to estimate direct and indirect effects between variables.

## Results

### Sample Characteristics

**FIGURE 1:**
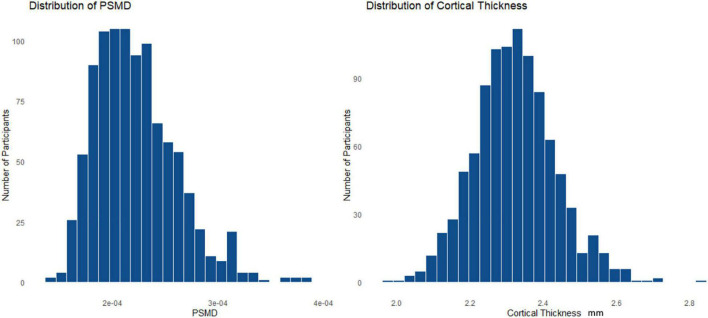
Histogram **(A)** is showing the distribution of the peak width of skeletonized mean diffusivity (PSMD) and histogram **(B)** is representing the distribution of the cortical thickness throughout the whole cohort.

**FIGURE 2:**
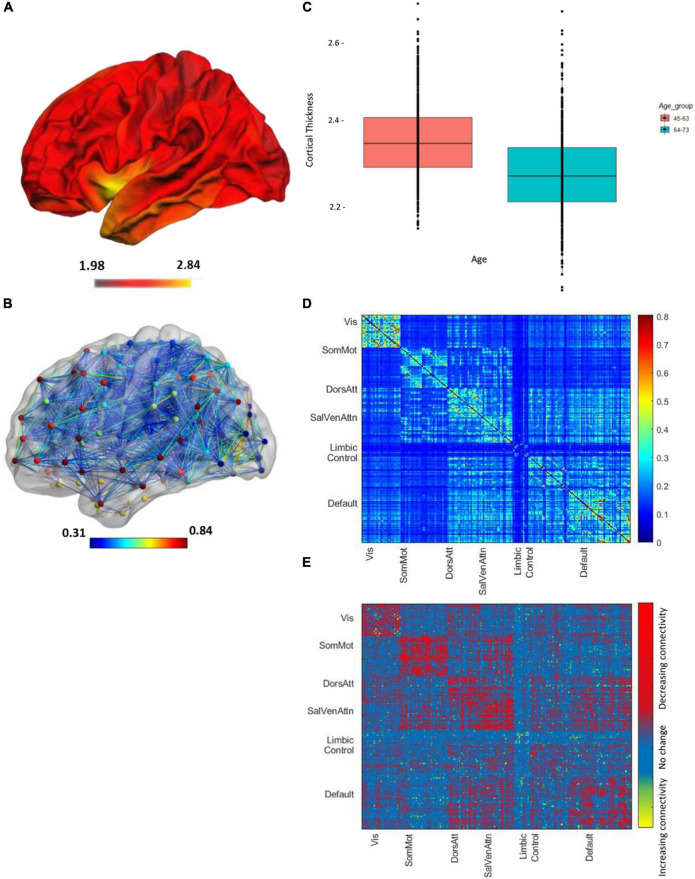
Imaging parameters of all the *n* = 976 participants, projected on a brain template in the Montreal National Institute (MNI) space. The color bar shows the mean cortical thickness (in mm). **(A)** Visualization of cortical thickness. **(B)** The boxplot shows the mean cortical thickness of between 45 and 63 years of age (red box) and subjects between 45 and 75 years of age (blue box). **(C)** Global functional connectivity network, the colors of nodes represent different networks the nodes belong to. The edge colors represent the strength of the correlation values. **(D)** Mean node-to-node correlation matrix of the whole cohort. Names along axes represent network labels and the color bar shows the correlation strength. **(E)** The difference map regions with decreasing and increasing connectivity for the younger vs. older age brains.

The demographics for the entire HCHS cohort and the median values of the cognitive test are shown in Table 1. Of 1,000 study participants, 24 study participants had to be excluded. During the visual examination of FLAIR images, one subject was directly excluded for severe imaging artifacts (*n* = 1). In addition, we excluded 23 subjects after the preprocessing due to bad image quality resulting in missing data in the connectivity matrices (*n* = 23). The remaining 976 subjects showed good image quality metrics assessed with the MRI-QC toolbox (Esteban et al., 2017).^5^ Median age was 63 years and 45.4% of participants were female. The median PSMD was 0.0002 [interquartile range (IQR) 0.0001], while global cortical thickness had a median of 2.319 (IQR 0.141). The distribution of the two structural parameters is shown in Figure 1; the cortical thickness for young as well as older participants of the cohort is additionally presented with a boxplot in Figure 2C. The mean FC throughout the whole cortex measured as the mean correlation between all the regions of all the participants was 0.362 (IQR = 0.04). The mean connectivity matrix of all the participants is shown in Figure 2D along with a difference map. Figure 2E shows the change in FC between younger and older participants of the cohort (see Figure 2). Within the resting-state networks, the default mode network showed a mean connectivity of 0.42 (IQR = 0.05), the dorsal network showed a mean connectivity of 0.42 (IQR = 0.08), the salience network showed a mean connectivity of 0.4 (IQR = 0.09), and the control network showed a mean connectivity of 0.38 (IQR = 0.09).

**TABLE 1:** Sample characteristics and image analysis results—subjects used for MRI analysis of the Hamburg City Health Study.

	Sample characteristics (*N* = 976)	Sample characteristics young 45–63 years (*N* = 490)	Sample characteristics old 64–75 years (*N* = 486)
Female sex (*n*, %)	443 (45.4%)	240 (49%)	203 (42%)
Age (years), median (IQR)	63 (13)	56 (7.75)	69 (5)
Education (years), median (IQR)	13 (4)	14 (5)	13 (4)
TMT B Score (s), median (IQR)	79 (37)	70 (32.5)	86 (42)
TMT A Score (s), median (IQR)	36 (16)	33 (13)	41 (18)
**Vascular risk factors**			
Current smoking (*n*, %)	155 (17.4%)	98 (20%)	71 (15%)
Diabetes* (*n*, %)	82 (9%)	38 (7%)	53 (11%)
BMI (kg/m^2^), median (IQR)	26.28 (5.6)	26.42 (5.8)	26.3 (5.5)
Hypertension** (*n*, %)	665 (72%)	283 (58%)	382 (79%)
**Structural MRI measures**			
PSMD, median (IQR)	0.000217 (0.00005)	0.0002 (0.00004)	0.00024 (0.00005)
Cortical thickness (mm) and median (IQR)	2.319 (0.141)	2.5 (0.12)	2.3 (0.14)

BMI, body mass index; IQR, interquartile range; mm, millimeter; PSMD, peak width of skeletonized mean diffusivity; TMT, Trail Making test. *Presence of diabetes was defined as blood glucose level > 126 mg/dl or a self-reported prevalence of diabetes. **Presence of hypertension was defined as blood pressure > one-fourth 140/90 mm Hg, intake of antihypertensive medication, or a self-reported prevalence of hypertension.

### Effects of Age on Brain Structure, Functional Connectivity, and Executive Function

**FIGURE 3:**
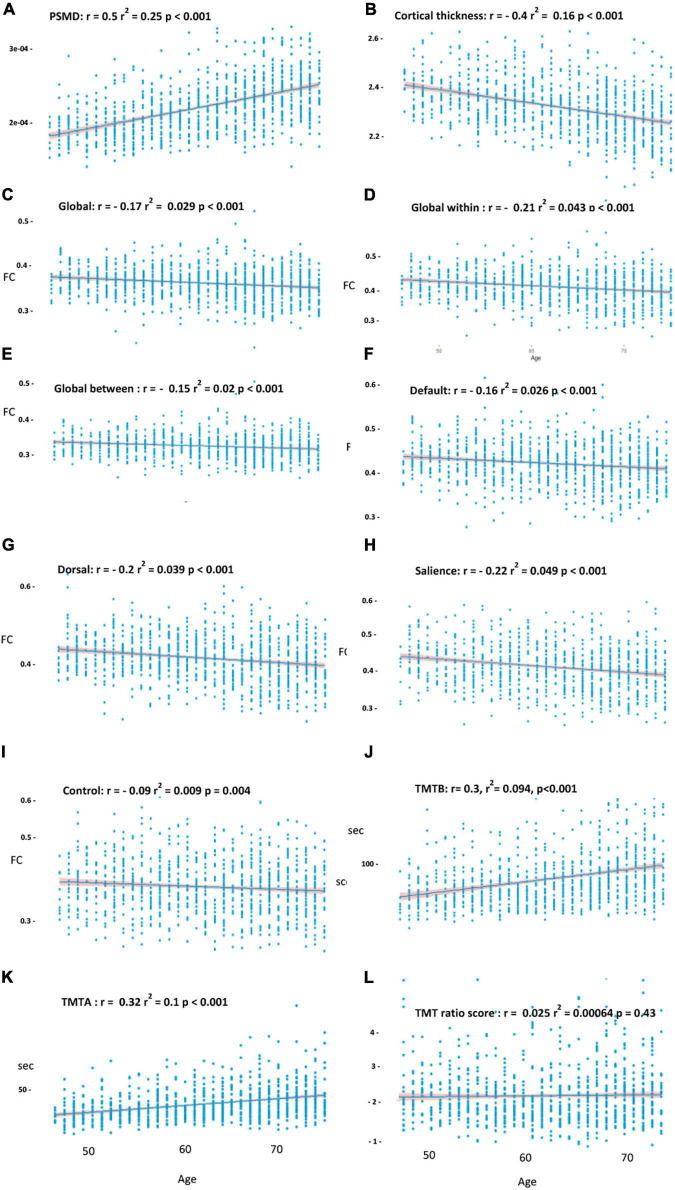
Effects of age on the PSMD **(A)** cortical thickness, **(B)** on global functional connectivity, **(C)** global intranetwork connectivity, **(D)** global between network connectivity, **(E)** within default, **(F)** dorsal, **(G)** salience, **(H)** control network, **(I)** the Trail Making Test B (TMTB), **(J)** TMTA, **(K)** and TMT ratio score **(L)**. Above *r* and *r*^2^ corresponds to the linear model before covariate inclusion. Shade around the regression line shows the 95% CI.

The univariate analyses revealed a significant effect of age on cortical thickness (*r*^2^ = 0.16, *r* = −0.4, *p* < 0.001), the PSMD (*r*^2^ = 0.25, *r* = 0.5, *p* < 0.001), global FC (*r*^2^ = 0.029, *r* = −0.17, *p* < 0.001), global intranetwork connectivity (*r*^2^ = 0.043, *r* = −0.21, *p* < 0.001), global between network connectivity (*r*^2^ = 0.02, *r* = −0.15, *p* < 0.001) as well as on default (*r*^2^ = 0.026, *r* = −0.16, *p* < 0.001), dorsal (*r*^2^ = 0.039, *r* = −0.2, *p* < 0.001), salience (*r*^2^ = 0.049, *r* = −0.22, *p* < 0.001), and control (*r*^2^ = 0.009, *r* = −0.09, *p* = 0.004) network connectivity, and on the TMTA score (*r* = 0.32, *r*^2^ = 0.1, *p* < 0.001) as well as on the TMTB score (*r*^2^ = 0.094, *r* = −0.3, *p* < 0.001), as shown in Figure 3. The TMTB/TMTA ratio score failed to demonstrate any association with age and was, therefore, excluded from further analysis. The univariate analysis of age and FC was additionally performed with different thresholds for the connectivity matrices, showing robust results throughout different thresholding, as shown in Supplementary Table 2. Of particular importance, since we used absolute values, we might not have measured age-related increase in between network connectivity, which correspond to reductions in the magnitude of negative or anticorelation. Therefore as a measure of control, we investigated the relationship between age and whole-global FC as well as separated into intra- and internetwork connectivity without absolute values and instead with signed matrix values throughout different thresholds. Supplementary Table 3 reports the results and Supplementary Figure 3 shows the mean FC matrices with signed values of all the participants. If no links or 30% of the weakest connections were excluded, a weak increase in internetwork connectivity could be observed in contrast to the analysis with absolute values and, as a result, no significant drop in whole global connectivity. This trend loses its significance when excluding 50% of the weakest connections and finally becomes negative when excluding 70% of the links.

### Effects of Brain Structure on Functional Connectivity

To analyze the effect of age, cortical thinning, and the PSMD on FC, we performed multiple linear regression analyses, adjusted for sex, and years of education. Therefore, we iteratively tested the model on a global scale and for each resting state network (default, dorsal, salience, and control). After adjusting the threshold for multiple comparisons, neither cortical thinning nor the PSMD predicted FC. Instead, the results of the linear model indicated a negative, but weak association between age and FC. Decreasing FC with increasing age was continuously observed across the entire brain (*r*^2^ = 0.034, *r* = −0.184, *p* < 0.001), for global intranetwork connectivity (*r*^2^ = 0.055, *r* = −0.23, *p* < 0.001), global between network connectivity (*r*^2^ = 0.036, *r* = −0.2, *p* < 0.001) as well as within the default (*r*^2^ = 0.029, *r* = −0.17, *p* < 0.001), dorsal (*r*^2^ = 0.039, *r* = −0.21, *p* < 0.001), and salience (*r*^2^ = 0.055, *r* = −0.234, *p* < 0.001) network. The correlation between age and FC within the control network did not exceed our statistical threshold (*r*^2^ = 0.014, *r* = −0.12, *p* = 0.04) and was, therefore, left out from further analysis. To investigate once again, if the results change using matrices with positive and negative values, the relationship between age, structural parameters, and FC was additionally evaluated without absolute values at a threshold of 50%. With the exception of internetwork connectivity, which no longer demonstrated any change with age, no significant differences could be observed compared to the analysis with absolute values (see Supplementary Table 4).

### Disconnected Subnetwork With Reduced Functional Connectivity With Higher Age

**FIGURE 4:**
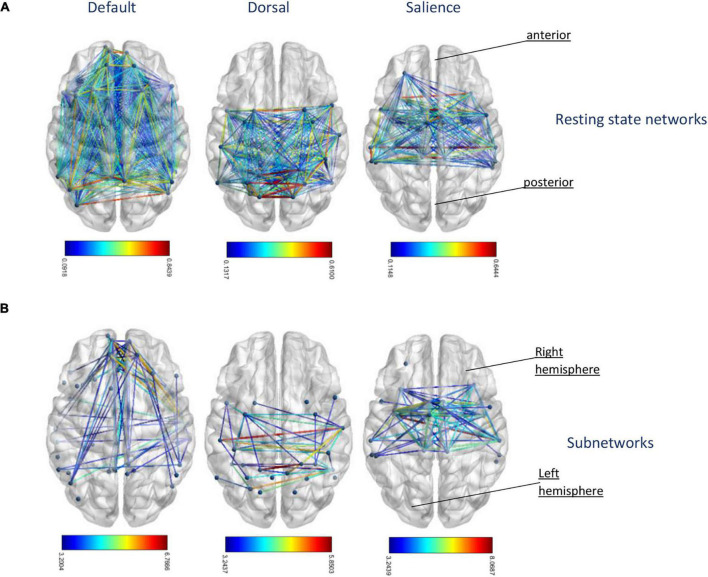
**(A)** Original resting-state networks of default, dorsal, and salience network from left to right. The colors of the edges represent different correlation strength of the mean connectivity matrix. **(B)** The corresponding extracted subnetworks network-based statistic (NBS) identified age-related disconnected functional subnetworks in default, dorsal, and salience network. The disconnections comprising this subnetwork correspond to pairs of nodes between which the resting-state times series was less correlated with increasing age. The colors of the edges represent test statistic results of the suprathresholded links, which exceed the threshold of *t*-value = 3.2.

The analysis using the NBS toolbox identified subnetworks significantly associated with age among the whole-brain network as well as within default, dorsal, and salience network, respectively (see Figure 4). Supplementary Table 1 provides a list of nodes comprising the disconnected subnetwork as well as their degree in this subnetwork. Subnetworks showing a significant decrease in connectivity with increasing age among default, dorsal, and salience network comprised 10, 13, and 36% of the connections of the original networks. Overall, subnetworks of connections with age-related reduced FC appeared to include a similar amount of inter- and intrahemispheric connections with no specific pattern as compared to the whole resting-state networks. In addition, we investigated whether there are areas within the resting-state network that exhibits an increase in connectivity with aging. However, based on the statistical threshold for multicomparison, we did not find any connected graph components with the NBS toolbox that showed increasing connectivity with age. Since the threshold of 50% of the connectivity matrices we used for the NBS toolbox is arbitrary, we repeated our subnetwork extraction with non-thresholded matrices. Despite small variation in the total number of regions, subnetworks extracted from thresholded and unthresholded matrices were highly consistent in terms of the number of links and regions involved (data not shown).

### Mediation Analysis

**FIGURE 5:**
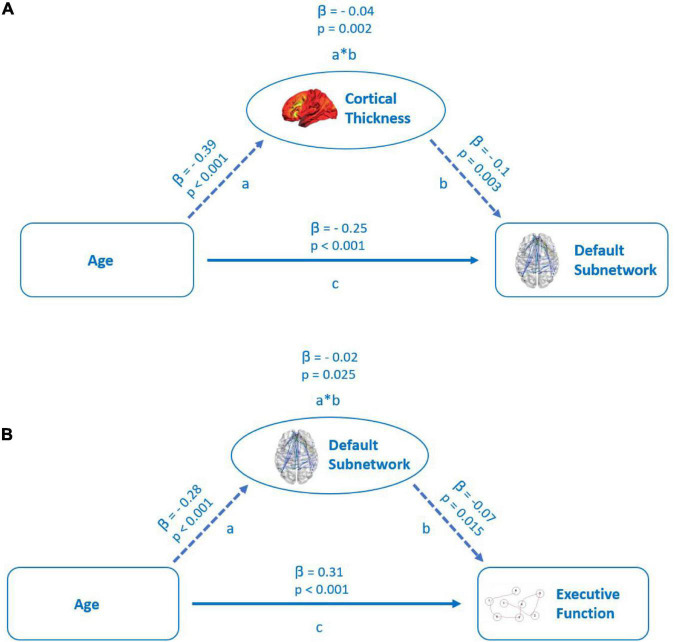
**(A)** Mediation of the relation between age and functional connectivity in default subnetwork by cortical thickness. Path a: relationship between age and cortical thickness. Path b: relationship between cortical thickness and default subnetwork connectivity. Path c: direct relationship between age and default subnetwork connectivity. **(B)** Mediation of the relation between age and executive cognitive function (TMTB) by functional connectivity of the default subnetwork. Path a: relationship between age and default subnetwork connectivity. Path b: relationship between default subnetwork connectivity and executive cognitive function. Path c: direct relationship between age and executive cognitive function. Both the models represent standardized beta coefficients and the respective *p*-values.

To assess whether structural parameters were mediators of the relation between age and FC, we performed mediation analyses of model one with age as a predictor of FC of our extracted subnetworks and the PSMD as well as cortical thickness as possible mediators between age and the outcome variables global, default, dorsal, and salience subnetworks mean connectivity (see Figure 5A). The mediators were modeled as operating in parallel and, thus, each mediator was a covariate for the others. In model two, global network and default, dorsal, and salience subnetwork mean connectivity were examined as mediators operating in parallel as well between the predictor age and the TMTB or the TMT A score as outcome variables (see Figure 5B). Results of this analysis are given below (see Table 2).

**TABLE 2:** Significant results after multiple testing corrections for the multiple regression as well as the mediation analysis, representing unstandardized coefficient estimate (beta), SE, standardized estimate (standard beta), the *p*-value, and the *r*^2^ value.

Significant results					
Variables	Beta	Std. error	Std. beta	*p*-Value	*R* ^2^
**Alteration of functional connectivity with age**					
Global mean connectivity	–0.0007	0.0002	–0.14	*p* < 0.001	0.034
Global mean within network connectivity	–0.0009	0.0002	–0.17	*p* < 0.001	0.049
Global mean between network connectivity	–0.0006	0.0002	–0.14	*p* < 0.001	0.027
Mean default connectivity	–0.0006	0.0002	–0.11	*p* = 0.005	0.029
Mean dorsal connectivity	–0.001	0.0003	–0.17	*p* < 0.001	0.039
Mean salience connectivity	–0.002	0.0003	–0.2	*p* < 0.001	0.055
**Mediation model one**					
Total effect	–0.004	<0.0001	–0.29	*p* < 0.001	–
Direct effect	–0.003	<0.0001	–0.24	*p* < 0.001	–
Indirect effect	–0.001	<0.0001	–0.04	*p* = 0.002	–
**Mediation model two**					
Total effect	1.365	0.138	0.31	*p* < 0.001	–
Direct effect	1.234	0.142	0.28	*p* < 0.001	–
Indirect effect	0.101	0.045	0.02	*p* = 0.025	–

In the first model, cortical thickness had a significant, but small (β = −0.04) partial mediating effect between age and FC in the default subnetwork, but not in other subnetworks (see Figure 5A). The PSMD had no significant mediating effect on the relationship between age and FC within the resting-state subnetworks.

Of all the subnetworks, only FC of the default subnetwork emerged as a significant, but weak partial mediator (β = 0.02) between age and the TMTB score (see Figure 5B), while FC in no other subnetwork had a significant mediating effect on the TMTB score. We did not observe any mediation effects of subnetworks between increasing age and the TMTA score outcomes. Even though not part of our hypothesis, we tested in an exploratory analysis the mediation effect of cortical thickness between age and the TMTB score in a separate model. Cortical thickness showed no significant mediation effects (see Supplementary Figure 1).

## Discussion

We studied the influence of age, microstructural white matter damage, and cortical thinning on FC and executive cognitive function in a healthy population at increased cardiovascular risk. We identified a significant association of age with all the structural and functional imaging parameters and cognition, although the association between age and FC was weak. However, neither the PSMD as a measure of diffuse white matter microstructural alteration nor cortical thinning revealed any association with FC when adjusted for age. After extracting subnetworks consisting exclusively of connections that showed a decreased connectivity with age, we discovered a small but significant mediation effect of global cortical thickness on the relation between age and FC in the default subnetwork. Finally, FC of the default subnetworks showing an age-associated decline had a weak but significant mediating effect on relation between age and executive cognitive function measured by the TMTB.

Age-related decline of brain function likely is the consequence of multiple biological factors, leading to structural and functional alterations, including cortical thinning, white matter injury, and loss of functional coordination between regions. In line with this assumption, with advancing age, we observed a linear pattern of increasing microstructural white matter damage (i.e., the increasing PSMD), increasing cortical thinning, and a weak decrease in FC. Moreover, older age was associated with worse psychomotor speed and executive function as measured by the TMTA and the TMTB. These findings confirm those previous studies showing age-dependent linear pattern of changes of resting-state FC, structural damage, and cognitive performance (Onoda et al., 2012; Veldsman et al., 2020). Some reports of non-linear age-related patterns demonstrate an accelerated decline in structural measurements (Fjell and Walhovd, 2010) and FC (Betzel et al., 2014) in old age or even a non-linear increase of FC in middle adulthood (Staffaroni et al., 2018) in particular concerning the global connectivity between networks (Betzel et al., 2014; Chan et al., 2014). We could only monitor an increase in connectivity between networks in the additional control analysis of matrices with negative and positive correlations that were subjected to a low threshold or no thresholding at all. The cross-sectional design and the limited range of age including only participants between 46 and 75 years of age may have reduced the possibility of detecting stronger or opposing age-related trends and, thus, further efforts are required to determine the shape of progression of imaging measurements over lifetime. Regarding FC in particular, we reproduced the overall findings in literature of age-related FC decrements, which seem to preferentially affect default (Damoiseaux et al., 2008; Dey et al., 2016), dorsal network (Tomasi and Volkow, 2012; Staffaroni et al., 2018), and salience network (Vidal-Piñeiro et al., 2014; Ng et al., 2016). However, the negative influence of age on FC identified in this study was only weak, which might indicate resilience of FC to aging across a wide age range. Other studies observed a somewhat stronger relationship between age and FC (Chan et al., 2014; Madden et al., 2017). This discrepancy can possibly be explained by the fact that their sample comprised a significantly higher age range and a higher age maximum. Stronger associations between age and FC are likely to be seen when comparing the brain of average 20-year-old and the brain of average 80-year-old (Betzel et al., 2014; Madden et al., 2017). Another possible explanation concerns the character of the studied cohort itself. Considering the expectation that cardiovascular risk factors are more prevalent in older adults, the selection may have a particular impact on younger middle-aged participants because they disproportionately represent individuals with poor cardiovascular health compared with age-matched individuals in other more healthier samples or the general population. Due to the sensitivity of FC to cardiovascular health, this may ultimately have reduced the age differences in FC in the current sample.

In our multiple regression model, we found no direct effect of the PSMD or cortical thinning on FC neither on global FC nor on the connectivity of the resting-state networks when corrected for age. Aging, on the other hand, was independent from the structural imaging measures associated with different degrees of lower average FC throughout the whole brain, global intra- as well as internetwork connectivity, and within three of the four resting-state networks, indicating a broadly distributed but not uniform age-related decline. In our multiple regression model, we did not detect an effect of age on the control network, even though a decrease of FC in this network has been reported (Betzel et al., 2014). The fact that no change could be observed in our sample can again probably be attributed to the lower median age and smaller age range as compared to other studies. A decrease of FC in the frontoparietal control network may only appear at a later point in time or only with more severe structural damage.

Nevertheless, our findings suggest an independent influence of age on structural brain changes and FC. While recent studies have found that FC tends to be reduced globally and within resting-state networks with increasing age, the relationship between structural white matter damage and FC seems to be less clear. According to current knowledge, temporospatial organization of neuronal activity in the brain is supported and constrained by the anatomy of axonal projections that form structural connections between both the adjacent and remote brain areas (van den Heuvel and Sporns, 2019; Suárez et al., 2020). In this context, the “disconnection hypothesis” indicates that compromised integrity of these white matter pathways, e.g., WMH or altered MD, inflicts a deficit in FC. Yet, even though a variety of studies with clinically and/or radiologically manifest patients with CSVD report a relationship between white matter lesions and FC (Yi et al., 2012, 2015; Schaefer et al., 2014; Wang et al., 2019), several studies with cognitively normal and healthy individuals with low white matter lesion volume found no relationship between structural damage and FC (Madden et al., 2017; Staffaroni et al., 2018). At this point, we may conclude that a low lesion volume or small change in diffusion pattern does not affect the pattern of synchronous neuronal activation and that the measured decline in FC in our population is driven by other factors. However, in most articles, as also in this study, the extent of white matter damage has been quantified by total normalized values of the whole brain. If we assume that lesions in functionally silent brain areas can be compensated more easily by rerouting information through alternative redundant pathways, while even small lesions in functionally relevant hubs are likely to be associated with significant functional limitations. In this case, total lesion volume or the global PSMD may have insufficient explanatory value regarding the impairment of neuronal communication.

Given these considerations, the approach of measuring mean cortical thickness to reflect neurodegeneration can also be viewed critically, since the impact of cortical atrophy on FC may vary for different regions. Age-dependent cortical atrophy has been observed in several studies (Fjell et al., 2009; Azevedo et al., 2019) and could already be linked to FC (Kim and Lee, 2011; Van Tol et al., 2014). Cortical thickness has also been associated with white matter lesions (Mayer et al., 2020) and conceptualized as mechanism underlying this association, secondary cortical degeneration through white matter tracts damaged by WMH (Thong et al., 2013) was suggested. Indeed, several studies show that cortical atrophy in different regions is shaped by profiles and degree of underlying white matter connectivity and their corresponding lesions (Reijmer et al., 2017; Mayer et al., 2020). Thus, the impact on FC might differ according to the degree of atrophy in specific regions and the strategic importance of these affected regions.

We identified subnetworks of significant age-related decreased FC among the studied networks. These subnetworks comprised between 10 and 36% of the total network connections, affecting both the hemispheres equally and including both the inter- and intrahemispheric connections. These results suggest that the effect of age on FC does not show any specific regional pattern, but can be observed in a small fraction of functional connections across the entire brain. Overall, the relationship between cortical thickness and FC observed in this study was weak. This again might be related to the fact that broad structural measurements such as mean cortical thickness are too rough to detect subtle and more localized alterations in functional brain networks.

The association between age and executive function was in part mediated by FC among the network parts of the default subnetwork that were significantly altered with age. Executive function has been reported to be particularly sensitive to decline with increasing age (Wecker et al., 2000; Verhaeghen and Cerella, 2002) and associations of age-related decreased default mode activity and executive function have been observed in previous studies (Damoiseaux et al., 2008). In theory, the default mode network is deactivated when the person engages in a task and aging might impair this deactivation (Dey et al., 2016) and attenuate the function of the default mode network as a network-hub, leading to lower executive cognitive performance. Our results suggest that decline of executive cognitive performance with age might at least to some extent results from altered communication between brain regions representing nodes of the default mode network. Again, the association between disturbed FC and executive function was weak, which may relate to the fact that cognitive function is complex and the idea of relating specific cognitive functions to simple connectivity measures between specific brain regions within a single network is likely to fall short (Chan et al., 2014). In addition, our mediation analysis showed no significant mediator effect of FC on the TMTA, only a trend with the connectivity of the default subnetwork. However, because the TMTB/TMTA ratio score failed to demonstrate any alterations with an increasing age and was, therefore, not considered for the mediation model, we cannot exclude a potential influence of psychomotor speed on our measurement of executive cognitive function.

This study relies on a large number of participants and modern methodological approaches with multilevel analysis. Nevertheless, we included only a limited number of structural, functional, and cognitive variables. The total variance in FC shared with age; both the structural parameters and the covariates sex and education were ranging from ∼3 to 6% (by *r*^2^) meaning that most of the person-specific variation in FC is explained by parameters not included in our model. Our ability to identify mediating influences of structural alterations on age-functional connectivity relationships may be compromised by the relatively small relationships between age and FC itself. Furthermore, it is likely that the observed functional decline in dorsal and salience networks is also reflected by changes in cognitive function, which, however, are not monitored by the TMTA or the TMTB. Finally, this is a cross-sectional study in which dynamic developments with age can only be captured to a limited extent and which does not allow inference about causality of observed association.

In summary, we report an age-accompanied linear increase in markers of structural brain damage and a weak linear decrease in FC and executive cognitive function in a large population-based cohort of middle-aged to older participants with vascular risk factors. Our results indicate that alterations in FC with increasing age are not uniform across all the networks and affected networks show a limited number of broadly distributed connections with age-related functional decrease. The PSMD, as a marker of microstructural white matter damage, had no mediating effect between age and FC changes, but decreased cortical thickness had such an effect, albeit weak. Finally, FC of the age-dependent parts of the default mode network showed a weak association with performance in a test of executive function. Altogether, these findings suggest a potential although weak role of age-related cortical brain changes in mediating the effects of higher age on functional brain connectivity and weak role of age-related functional brain connectivity in selected brain networks on impaired cognitive function.

## Data Availability Statement

Anonymized data of the analysis not published within this article will be made available on reasonable request from any qualified investigator after evaluation of the request by the Steering Board of the HCHS. Requests to access these datasets should be directed to RT, r.twerenbold@uke.de.

## Ethics Statement

The studies involving human participants were reviewed and approved by Landesärztekammer Hamburg (State of Hamburg Chamber of Physicians, PV5131). The patients/participants provided their written informed consent to participate in this study.

## Author Contributions

MS: conceptualization, formal analysis, visualization, and writing – original draft. MS, ES, MP, CMay, and BF: data curation. MS, ES, MP, CMal, BF, CMay, UH, RT, JF, SK, JG, CG, GT, and BC: investigation and writing – review and editing. MS, ES, and CMal: methodology and software. GT and BC: supervision. All authors described contributions to the manuscript using the CRediT contributor role taxonomy and approved the submitted version.

## Conflict of Interest

The authors declare that this study received funding from Amgen, Astra Zeneca, Bayer, Badischen Anilin & Sodafabrik (BASF), Novartis, Pfizer, Schiller, Siemens, and Unilever. The funders were not involved in the study design, collection, analysis, interpretation of data, the writing of this article, or the decision to submit it for publication.

## Publisher’s Note

All claims expressed in this article are solely those of the authors and do not necessarily represent those of their affiliated organizations, or those of the publisher, the editors and the reviewers. Any product that may be evaluated in this article, or claim that may be made by its manufacturer, is not guaranteed or endorsed by the publisher.

## Footnotes

1
http://fmriprep.org

2
https://github.com/ANTsX/ANTs

3
https://surfer.nmr.mgh.harvard.edu

4
https://xcpengine.readthedocs.io

5
https://mriqc.readthedocs.io/en/stable/
