# Supplementary figures and images for: Association of Age and Structural Brain Changes With Functional Connectivity and Executive Function in a Middle-Aged to Older Population-Based Cohort
Source: Front Aging Neurosci. 2022 Feb 25;14:782738. doi: 10.3389/fnagi.2022.782738 (PMC8916110; doi:10.3389/fnagi.2022.782738)

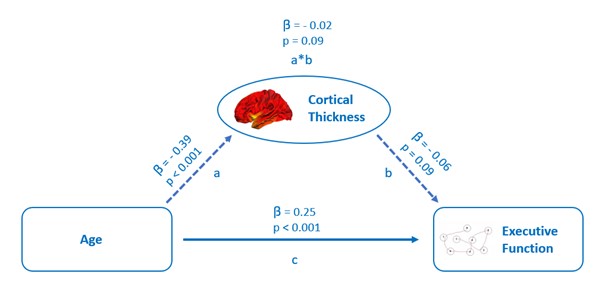

Supplement: Supplementary file 1 [file Image_1.jpg]

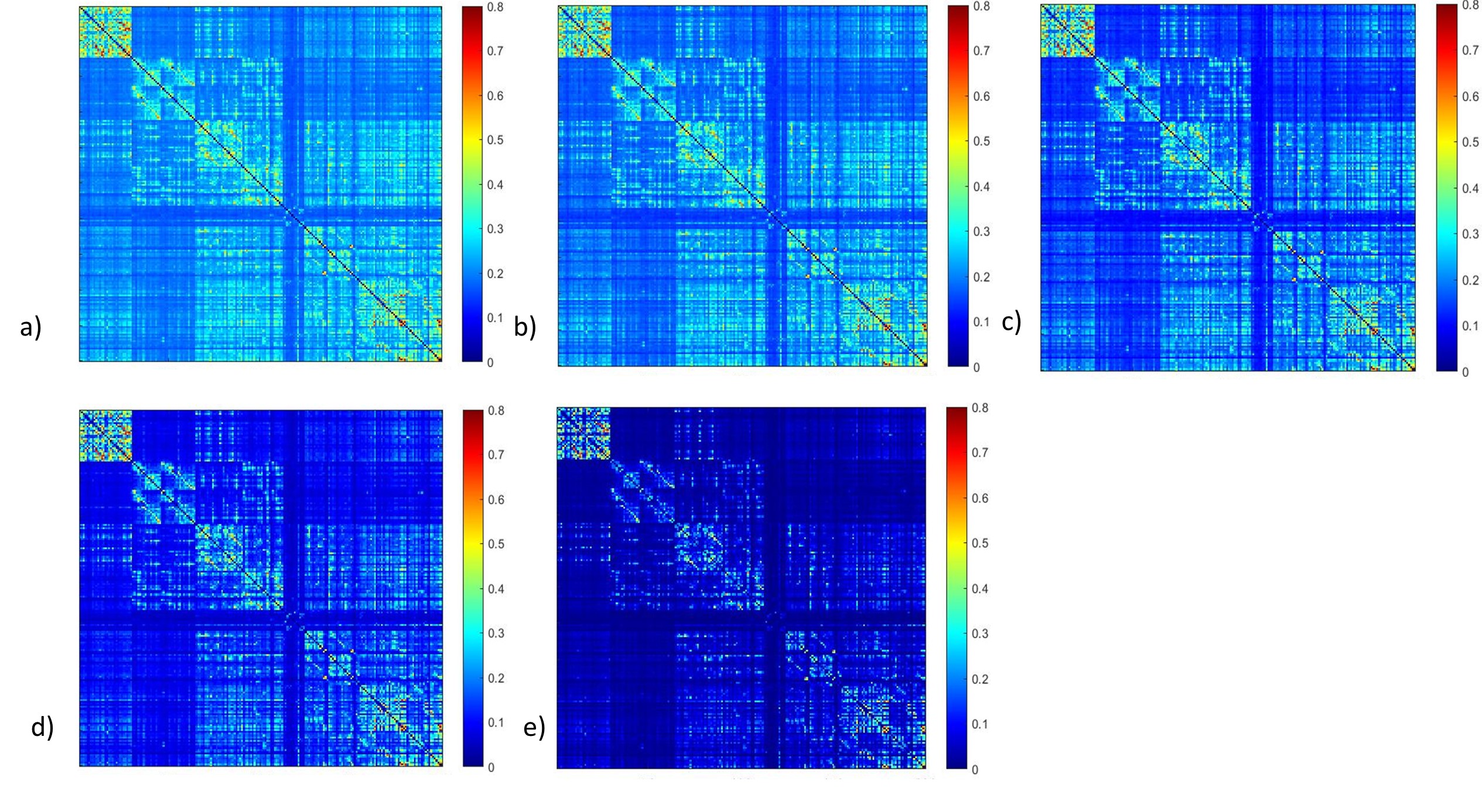

Supplement: Supplementary file 2 [file Image_2.jpg]

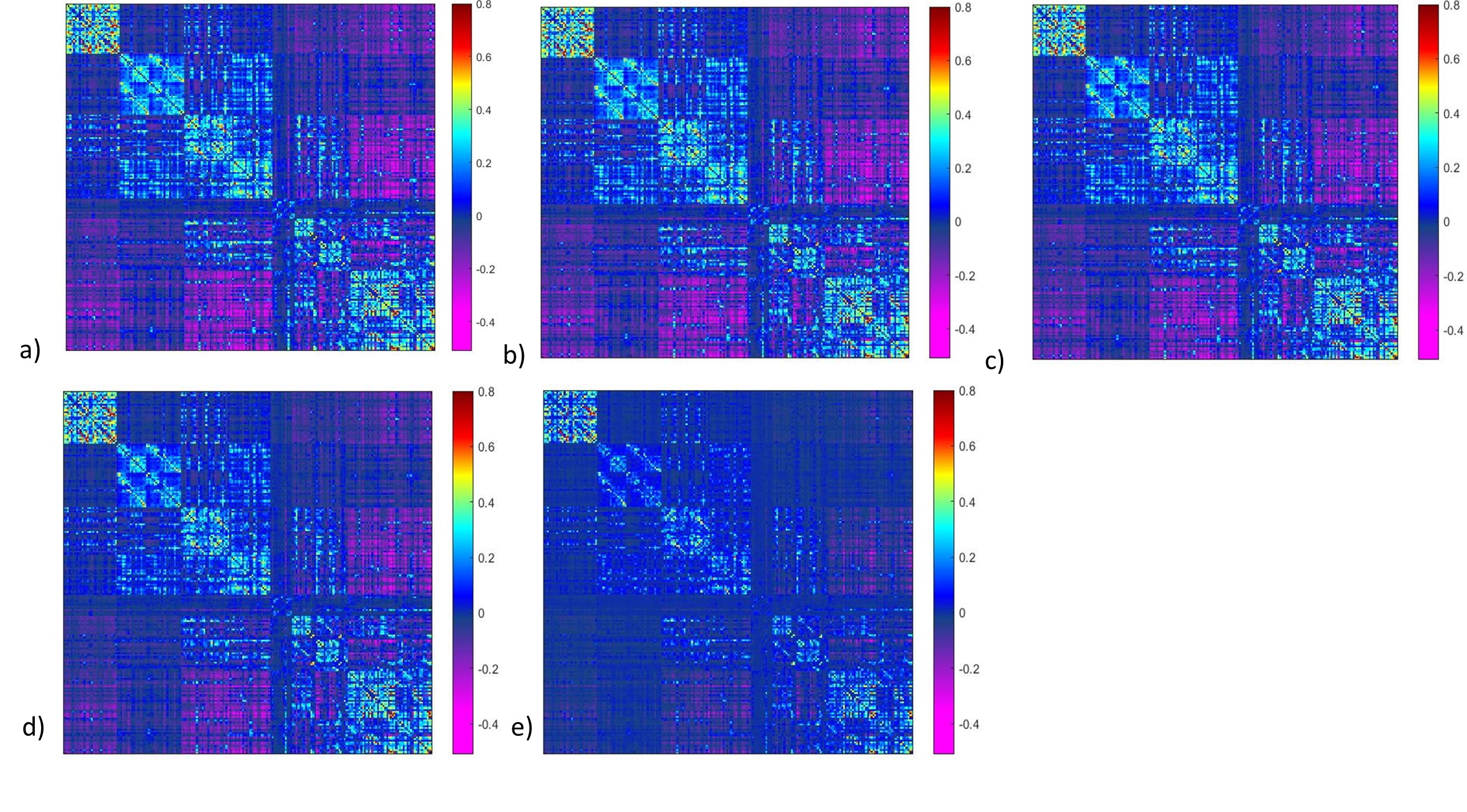

Supplement: Supplementary file 3 [file Image_3.jpg]
