# Supplementary material for: Association of Age and Structural Brain Changes With Functional Connectivity and Executive Function in a Middle-Aged to Older Population-Based Cohort
Source: Front Aging Neurosci. 2022 Feb 25;14:782738. doi: 10.3389/fnagi.2022.782738 (PMC8916110; doi:10.3389/fnagi.2022.782738)
Supplement: Supplementary file 4 [file Table_1.docx]

| **Abbreviations** | **MNI Coordinates** | **Degree** |
| --- | --- | --- |
| ***Default Subnetwork*** | | |
| LH_Default_Temp_1 | -47 8 -33 | 1 |
| LH_Default_Temp_2 | -60 -19 -22 | 2 |
| LH_Default_Temp_3 | -56 -6 -12 | 2 |
| LH_Default_Temp_4 | -58 -30 -4 | 2 |
| LH_Default_Temp_8 | -57 -54 28 | 2 |
| LH_Default_Temp_9 | -46 -66 38 | 7 |
| LH_Default_PFC_1 | -35 20 -13 | 6 |
| LH_Default_PFC_2 | -6 36 -10 | 14 |
| LH_Default_PFC_3 | -46 31 -7 | 1 |
| LH_Default_PFC_4 | -12 63 -6 | 5 |
| LH_Default_PFC_6 | -6 44 7 | 13 |
| LH_Default_PFC_7 | -8 59 21 | 15 |
| LH_Default_PFC_8 | -6 30 25 | 2 |
| LH_Default_PFC_9 | -11 47 45 | 12 |
| LH_Default_PFC_12 | -24 25 49 | 1 |
| LH_Default_PCC_1 | -11 -56 13 | 1 |
| LH_Default_PCC_2 | -5 -55 27 | 2 |
| LH_Default_PCC_3 | -4 -31 36 | 3 |
| RH_Default_Par_2 | 54 -50 28 | 4 |
| RH_Default_Par_3 | 51 -59 44 | 1 |
| RH_Default_Temp_1 | 47 13 -30 | 5 |
| RH_Default_Temp_2 | 61 -13 -21 | 15 |
| RH_Default_Temp_3 | 55 -6 -10 | 2 |
| RH_Default_Temp_4 | 63 -27 -6 | 6 |
| RH_Default_Temp_5 | 52 -31 2 | 2 |
| RH_Default_PFCv_1 | 51 28 0 | 1 |
| RH_Default_PFCm_1 | 5 37 -14 | 12 |
| RH_Default_PFCm_2 | 8 42 4 | 14 |
| RH_Default_PFCm_3 | 6 29 15 | 3 |
| RH_Default_PFCm_4 | 8 58 18 | 11 |
| RH_Default_PFCm_5 | 15 46 44 | 9 |
| RH_Default_PFCm_7 | 23 24 53 | 2 |
| RH_Default_PCC_2 | 7 -49 31 | 8 |
| RH_Default_PCC_3 | 6 -58 44 | 1 |
| ***Dorsal Subnetwork*** | | |
| LH_DorsAttn_Post_1 | -43 -48 -19 | 8 |
| LH_DorsAttn_Post_2 | -57 -60 -1 | 1 |
| LH_DorsAttn_Post_3 | -26 -70 38 | 3 |
| LH_DorsAttn_Post_4 | -54 -27 42 | 4 |
| LH_DorsAttn_Post_5 | -41 -35 47 | 5 |
| LH_DorsAttn_Post_6 | -33 -49 47 | 2 |
| LH_DorsAttn_Post_7 | -17 -73 54 | 1 |
| LH_DorsAttn_Post_8 | -29 -60 59 | 2 |
| LH_DorsAttn_Post_10 | -17 -53 68 | 3 |
| LH_DorsAttn_FEF_1 | -31 -4 53 | 7 |
| LH_DorsAttn_FEF_2 | -22 6 62 | 1 |
| LH_DorsAttn_PrCv_1 | -48 6 29 | 3 |
| RH_DorsAttn_Post_1 | 50 -53 -15 | 14 |
| RH_DorsAttn_Post_3 | 59 -16 34 | 8 |
| RH_DorsAttn_Post_4 | 46 -38 49 | 4 |
| RH_DorsAttn_Post_5 | 41 -31 46 | 5 |
| RH_DorsAttn_Post_7 | 34 -48 51 | 4 |
| RH_DorsAttn_Post_8 | 26 -61 58 | 1 |
| RH_DorsAttn_Post_9 | 8 -56 61 | 2 |
| RH_DorsAttn_Post_10 | 21 -48 70 | 5 |
| RH_DorsAttn_FEF_1 | 34 -4 52 | 3 |
| RH_DorsAttn_PrCv_1 | 52 11 21 | 1 |
| ***Salience Subnetwork*** | | |
| LH_SalVentAttn_ParOper_1 | -56 -40 20 | 5 |
| LH_SalVentAttn_ParOper_2 | -61 -26 28 | 5 |
| LH_SalVentAttn_ParOper_3 | -60 -39 36 | 1 |
| LH_SalVentAttn_FrOper_1 | -39 -4 -4 | 13 |
| LH_SalVentAttn_FrOper_2 | -33 20 5 | 9 |
| LH_SalVentAttn_FrOper_3 | -39 1 11 | 10 |
| LH_SalVentAttn_FrOper_4 | -51 9 11 | 1 |
| LH_SalVentAttn_Med_1 | -6 9 41 | 13 |
| LH_SalVentAttn_Med_2 | -11 -35 46 | 10 |
| LH_SalVentAttn_Med_3 | -6 -3 65 | 9 |
| RH_SalVentAttn_TempOccPar_2 | 60 -39 17 | 1 |
| RH_SalVentAttn_TempOccPar_3 | 60 -26 27 | 14 |
| RH_SalVentAttn_PrC_1 | 51 4 40 | 1 |
| RH_SalVentAttn_FrOper_2 | 46 -4 -4 | 11 |
| RH_SalVentAttn_FrOper_3 | 36 24 5 | 8 |
| RH_SalVentAttn_FrOper_4 | 43 7 4 | 12 |
| RH_SalVentAttn_Med_1 | 7 9 41 | 12 |
| RH_SalVentAttn_Med_2 | 11 -36 47 | 8 |
| RH_SalVentAttn_Med_3 | 8 3 66 | 9 |

**Table 3**: lists the nodes comprising the disconnected subnetwork as well as their degree in this subnetwork and anatomical abbreviation.
