# Supplementary material for: Association of Age and Structural Brain Changes With Functional Connectivity and Executive Function in a Middle-Aged to Older Population-Based Cohort
Source: Front Aging Neurosci. 2022 Feb 25;14:782738. doi: 10.3389/fnagi.2022.782738 (PMC8916110; doi:10.3389/fnagi.2022.782738)
Supplement: Supplementary file 5 [file Table_2.docx]

***Table 4 (Supplements)***

**Table 4:** Results of univariate analysis of the effects of age on FC through different thresholds excluding from 0 to 90 % of the weakest links, representing the standard estimate (std. Beta), the p-value and the r^2^ value.

|  | **Threshold 0** | **Threshold 30%** | **Threshold 50%** | **Threshold 70%** | **Threshold 90%** |
| --- | --- | --- | --- | --- | --- |
| ***Std. Beta*** |  |  |  |  |  |
| Mean Connectivity | -0.16 | -0.17 | -0.17 | -0.21 | -0.2 |
| Global mean within network connectivity | -0.2 | -0.2 | -0.2 | -0.2 | -0.19 |
| Global mean between network connectivity | -0.13 | -0.14 | -0.15 | -0.16 | -0.19 |
| Default | -0.15 | -0.16 | -0.16 | -0.18 | -0.17 |
| Dorsal | -0.17 | -0.2 | -0.2 | -0.2 | -0.2 |
| Salience | -0.23 | -0.23 | -0.22 | 0.22 | 0.2 |
| Control | -0.05 | -0.67 | -0.095 | -0.12 | -0.14 |
| ***p-Value*** |  |  |  |  |  |
| Mean Connectivity | <0.001 | < 0.001 | < 0.001 | < 0.001 | < 0.001 |
| Global mean within network connectivity | <0.001 | < 0.001 | < 0.001 | < 0.001 | < 0.001 |
| Global mean between network connectivity | < 0.001 | < 0.001 | < 0.001 | < 0.001 | < 0.001 |
| Default | < 0.001 | < 0.001 | <0.001 | <0.001 | < 0.001 |
| Dorsal | < 0.001 | < 0.001 | < 0.001 | < 0.001 | <0.001 |
| Salience | < 0.001 | < 0.001 | < 0.001 | < 0.001 | < 0.001 |
| Control | 0.15 | 0.04 | < 0.003 | < 0.001 | < 0.001 |
| ***R^2^*** |  |  |  |  |  |
| Mean Connectivity | 0.03 | 0.03 | 0.03 | 0.04 | 0.04 |
| Global mean within network connectivity | 0.04 | 0.04 | 0.04 | 0.04 | 0.04 |
| Global mean between network connectivity | 0.02 | 0.02 | 0.02 | 0.03 | 0.04 |
| Default | 0.02 | 0.02 | 0.03 | 0.03 | 0.03 |
| Dorsal | 0.03 | 0.04 | 0.04 | 0.04 | 0.04 |
| Salience | 0.05 | 0.05 | 0.05 | 0.05 | 0.04 |
| Control | 0.0021 | 0.005 | 0.009 | 0.014 | 0.02 |
