# Supplementary material for: Association of Age and Structural Brain Changes With Functional Connectivity and Executive Function in a Middle-Aged to Older Population-Based Cohort
Source: Front Aging Neurosci. 2022 Feb 25;14:782738. doi: 10.3389/fnagi.2022.782738 (PMC8916110; doi:10.3389/fnagi.2022.782738)
Supplement: Supplementary file 6 [file Table_3.docx]

|  | **Threshold 0** | **Threshold 30%** | **Threshold 50%** | **Threshold 70%** | **Threshold 90%** |
| --- | --- | --- | --- | --- | --- |
| ***Std. Beta*** |  |  |  |  |  |
| Global mean connectivity | 0.03 | 0.02 | -0.14 | -0.16 | -0.13 |
| Global mean within network connectivity | -0.22 | -0.22 | -0.2 | -0.18 | -0.2 |
| Global mean between network connectivity | 0.16 | 0.17 | -0.04 | -0.1 | 0.035 |
| ***p-Value*** |  |  |  |  |  |
| Global mean connectivity | = 0.4 | = 0.5 | <0.001 | <0.001 | <0.001 |
| Global mean within network connectivity | <0.001 | <0.001 | <0.001 | <0.001 | <0.001 |
| Global mean between network connectivity | <0.001 | <0.001 | = 0.224 | = 0.002 | = 0.27 |
| ***R^2^*** |  |  |  |  |  |
| Global mean connectivity | 0.0007 | 0.0005 | 0.02 | 0.026 | 0.017 |
| Global mean within network connectivity | 0.05 | 0.05 | 0.04 | 0.03 | 0.04 |
| Global mean between network connectivity | 0.03 | 0.03 | 0.002 | 0.01 | 0.0013 |

**Table 5:** Results of univariate analysis of the effects of age on global functional connectivity through different thresholds excluding from 0 to 90 % of the weakest links in matrices without absolute values, representing the standard estimate (std. Beta), the p-value and the r^2^ value.
