# Supplementary material for: Association of Age and Structural Brain Changes With Functional Connectivity and Executive Function in a Middle-Aged to Older Population-Based Cohort
Source: Front Aging Neurosci. 2022 Feb 25;14:782738. doi: 10.3389/fnagi.2022.782738 (PMC8916110; doi:10.3389/fnagi.2022.782738)
Supplement: Supplementary file 7 [file Table_4.docx]

| **Significant Results** | | | | |  |
| --- | --- | --- | --- | --- | --- |
| **Variables** | **Beta** | **Std. Error** | **Std. Beta** | **p-Value** | **R^2^** |
| **Alteration of functional connectivity with age** | | | | |  |
| Global mean connectivity | - 0.0003 | 0.00006 | - 0.14 | p < 0.001 | 0.02 |
| Global mean within network connectivity | - 0.001 | 0.0002 | - 0.2 | p < 0.001 | 0.038 |
| Global mean between network connectivity | - 0.0001 | 0.0001 | - 0.04 | p = 0.22 | 0.002 |
| Mean Default connectivity | - 0.0009 | 0.0002 | - 0.15 | p < 0.001 | 0.022 |
| Mean Dorsal connectivity | - 0.0015 | 0.0003 | - 0.17 | p < 0.001 | 0.031 |
| Mean Salience connectivity | - 0.002 | 0.0003 | - 0.22 | p < 0.001 | 0.05 |
| **Mediation Model on** | | | | |  |
| Total Effect | - 0.004 | < 0.001 | - 0.29 | p < 0.001 | - |
| Direct Effect | - 0.003 | < 0.001 | - 0.23 | p < 0.001 | - |
| Indirect Effect | - 0.001 | < 0.001 | - 0.04 | p = 0.002 | - |
| **Mediation Model two** | | | | |  |
| Total Effect | 1.2 | 0.141 | 0.26 | p < 0.001 | - |
| Direct Effect | 1.1 | 0.15 | 0.23 | p < 0.001 | - |
| Indirect Effect | 0.11 | 0.046 | 0.02 | p = 0.017 | - |

**Table 6.** Significant results after multiple testing correction for the multiple regression as well as the mediation analysis, representing unstandardized coefficient estimate (Beta), the standard error, standardized estimate (std. Beta), the p-value and the r^2^ value calculated with matrices without absolute values.
